# COVID-19 Suicide Survivors—A Hidden Grieving Population

**DOI:** 10.3389/fpsyt.2020.626807

**Published:** 2020-12-21

**Authors:** Sara Pinto, Joana Soares, Alzira Silva, Rosário Curral, Rui Coelho

**Affiliations:** ^1^Psychiatry Service, Centro Hospitalar Universitário São João, Oporto, Portugal; ^2^Department of Clinical Neurosciences and Mental Health, Faculty of Medicine of the University of Oporto, Oporto, Portugal; ^3^Psychology Service, Centro Hospitalar Universitário São João, Oporto, Portugal

**Keywords:** complicated grief, suicide, suicide survivors, COVID-19, intervention, prevention

## Abstract

Present time has been dominated by the COVID-19 pandemic. People are grieving several non-death related situations: the loss of a job, of a status, of a role, of their life. Restrictive measures and uncertainty about the future makes individuals vulnerable to feelings of hopelessness and helplessness. Mental health support has been hindered and teams are reinventing themselves to reach people in need. Nevertheless, decompensation of previous psychiatric disorders, increasing levels of depression and anxiety, economical handicaps and fear of the infection, are prompting several cases of COVID-19 related suicides worldwide. Every suicide affects between 5 and 80 individuals, which are known as suicide survivors. Suicide grief is particularly challenging, with rates of complicated grief as high as 40%. Suicide survivors are at increased risk of developing depression, anxiety disorders and of suicidal behaviors. Moreover, feelings of guilt and shame, as well as social stigma, are major obstacles for them to reach form help. This article aims to review the existing literature on COVID-19 related suicides, complicated grief in suicide survivors and highlight modifiable risk factors for both conditions, as well as propose some public health measures to reduce the impact of the pandemic context on self-inflicted harm and its consequences on families, friends and the community. Obstacles to access to mental health support need to be overcome through the use of technology. Technicians should actively approach populations more vulnerable to develop suicidal ideation. Social media have the obligation to provide accurate an non-sensationalistic information. Families and friends should maintain social proximity, despite the need for physical distancing. When a suicide death occurs, police forces and health staff should be prepared to share the news with the family using an empathic and humane approach and providing psychological support. Funerals, memorials and other services should be held as much as possible. Closer contacts should be signalized and closely followed in order to detect the need for specific interventions. Help seeking behaviors should be promoted. Additionally, people should be educated on suicide and its impacts, in order to reduce stigma.

## Introduction

The infection by Sars-CoV2 has been classified as pandemic by the World Health Organization on the 12th March 2020. Since its detection in the Chinese city of Wuhan up until the time of writing this manuscript (30th October 2020), COVID-19 has already affected more than 46 million people worldwide and caused more than 1,100,000 deaths. In Portugal, as off today, more than 141 thousand people were infected and COVID-19 was responsible for 2,507 deaths.

As the virus spread, governments urged to impose aggressive measures, in order to mitigate transmission and prevent the collapse of the health system structure. Compulsory confinement was applied, working from home implemented, schools closed, visits to hospitals, hospices and other healthcare facilities suspended, non-urgent health procedures suspended, and non-essential economical activities closed. Although necessary and urgent, such measures had a major impact in people's everyday routines, jobs, economic status, social connections, self-care and leisure activities.

In Portugal, about 877 thousand people were set into layoff from their work (1), while the unemployment rate increased 22.1% [comparing with April/2019 (2)]. A similar scenario was observed worldwide. This drop in individual and family incomes posed as a major factor of individual a relational stress. Requests for help regarding food and medical supplies dramatically increased.

Another important factor was the compulsory or recommended lockdown people were subjected to—during the month of April, about half of the world population (3.9 billion people) were confined to their homes and households (3). This forced confinement, initially promoted as “social distancing,” led to a decrease in contacts and weakening of support networks. On the one hand, chronically ill patients, the elderly and the economically underprivileged people were deprived from their support and care structures, receiving help only for basic and instrumental daily needs (e.g., distribution of food and medical drugs, emergency medical assessment). Emotional and affective support was scarce, with a marked increase of feelings of loneliness and isolation. On the other hand, stress levels and conflicts increased among households, with families forced to interact on the same space, for 24 h a day, 7 days a week. Parents were both workers and teachers, as home schooling and remote working were implemented. A complete disruption of routines was observed in many cases, with longer working journeys and less quantity and quality of family and individual time. Despite living in a technological era, during periods of lockdown and promoted social distancing/isolation, it has been shown that artificial substitutes for social connection (such as video-calls, online group events) appear to exacerbate pre-existing feelings of disconnection. Furthermore, these substitutes will never manage to fulfill the role and status people have in their active daily living and live social events (4).

Alongside with social distancing and increased isolation, media coverage of the COVID crisis potentiated people's whole perception of risk, raising fear and anxiety (5).

In health care structures, professionals were dealing with an extreme reality—lack of individual protection material, insufficient technical support for severely ill patients, frequently working extra hours and being forced to postpone “non-urgent care.” This need to direct all available means to face the pandemic crisis led to a conscious neglect of patients with other types of pathology.

Mental health care was significantly affected—both scheduled appointments and urgent assessments. There was already plenty of evidence that mental health disorders, such as anxiety, depression substance abuse, as well as suicide attempts/completed suicides, have been increasing in the last years (6). Even before the COVID-19 pandemic, mental health services could not reach and adequately answer to the population in need. Given the current reality, it is expected that mental health care demands will increase—anxiety, depression and substance abuse are common reactions to major crisis (7). This insufficient response from healthcare structures, may potentiate the growth of a “mental health need curve,” that will last beyond the COVID-19 pandemic crisis (8, 9).

Extreme cases of social and economic overload, in a background of fragile mental health, bring together important risk factors for the appearance of suicidal ideation, suicide attempts and completed suicides. Suicide has long been considered a global public health problem, with about 800,000 people taking their lives each year (10). It is a complex and multidimensional phenomenon. However, nearly 90% of all suicides occur in individuals with a diagnosed mental health or substance-abuse disorder (11). Furthermore, suicide attempters frequently describe feelings of hopelessness, despair and social isolation as triggers for acting (12). According to WHO data, each suicide is accompanied by more than 20 suicide attempts (10).

It has been described that each suicide may affect between 5 nuclear family members and 80 relatives, friends and acquaintances (13). A meta-analysis by Andriessen et al. (14) found that 4.3% of people have experienced a suicide in the previous year, with 21,8% being exposed to suicide during their lifetime. This makes suicide a highly pervasive phenomenon, with the group of suicide survivors being one of the largest at risk communities for mental health disorders. Suicide survivors show increased risk of complicated grief, depressive and anxiety disorders, substance-abuse disorders and suicidal behavior (15).

Grieving suicide survivors are a particular group of individuals. Grief is the natural reaction to the loss of a significant one, comprehending emotional, psychological, physical and behavioral responses to the death (16). However, people grieving suicide also present an important component of shock and possible trauma, as well as feelings of abandonment, rejection, unacceptance and shame related to the circumstances of death (17, 18). The normative process of grief usually evolves from acute to integrated, being individuals able to return to their normal daily living and functional level. When some internal or external factor interferes with this process, complicated grief may occur. It has been shown that complicated grief is more frequent in suicide survivors than in people grieving other causes of death (19), affecting up to 40% of individuals in some samples (20). Besides all the common risk factors for complicated grief, suicide survivors are subjected to high levels of stigma, especially present as social disapproval, isolation and shunning (21). People grieving suicide also present higher levels of physical comorbidities, much related to the lack of selfcare and adoption of unhealthy lifestyles observed in the course of bereavement (22).

Given the high risk of psychiatric and physical disorders, suicide survivors should be closely followed and intervention initiated as soon as possible—the concept of postvention, used by Erlangsen and Pitman (22) aims at an early professional intervention, in order to prevent deleterious outcomes. It is mandatory to facilitate the access of this community to mental healthcare facilities, as well as to individual and group therapies. Due to the current reality, characterized by physical distancing and fear of the Sars-CoV2 infection, it is important to reframe the psychotherapeutic setting and adapt it to the online world.

At a time when the world is fully entering the second wave of COVID-19, with the prevision of new lockdown periods, reinforced social isolation and a new drop in economic activities, it is imperative to focus on suicide prevention and follow up of suicide survivors.

The aim of this article is to review the existing literature on COVID-19 related suicides, complicated grief in suicide survivors and highlight modifiable risk factors for both conditions, as well as propose some public health measures to reduce the impact of the pandemic context on self-inflicted harm and its consequences on families, friends and the community.

## The COVID-19 World and Increased Risk for Suicide

As briefly exposed in the introduction section, the world as we knew it has significantly changed as a result of the restrictions imposed to contain the COVID-19 pandemic. The interpersonal theory of suicide tells us that suicide, as a multidimensional phenomenon, may be the result of hindered belongingness and perceived burdensomeness, associated with an acquired capability for suicide (23). Forced lockdowns, canceled medical appointments, lack of social support, unemployment and fear for the future make several populations prone to suicide. Various studies were published regarding the impact of previous pandemics in suicide rates worldwide. Deaths by suicide increased in the United States of America during the 1918–1919 influenza pandemic (24), as well as in Hong Kong during the 2003 severe acute respiratory syndrome (SARS) epidemic—this latter increase was specially observed in elder people (25, 26). During the SARS outbreak, local increases in suicides were seen in the context of forced quarantines (e.g., Taipei hospital) (27). Longer durations of quarantine, frustration, boredom, insufficient information, inadequate supplies and fear of infection were identified as important risk factors for the occurrence of negative outcomes (28). Another study validated that a disproportionate increase in rates of psychological distress was seen in areas that adopted longer periods of quarantine (29), and higher rates of post-traumatic stress disorder (PTSD) observed in residents of high SARS-prevalent areas (30).

One of the main factors associated with the increased rates of suicide in previous pandemics has been unemployment. Unemployment by itself is associated with a 2–3-fold increased risk of death by suicide (31). The Global Financial Crisis was responsible for the loss of 30 million jobs and unemployment were responsible for 10,000 “economic suicides” between 2008 and 2010 in Europe and North America (32)—many of them preceded the actual rise in unemployment (33).

Regarding the present crisis, the International Monetary Fund has predicted it will constitute the steepest economic downturn since the Great Recession (34). Developing and less developed countries are experiencing more extensive crises that developed ones—many small and medium sized businesses are being disrupted, going bankrupt and closing (35). Studies modeling the effect of present unemployment predictions on suicide increase point toward more 2,135 to 9,570 completed suicides in the context of COVID-19 crisis (36). Other authors target for a 3.3–8.4% increase in suicides in the US, over the period of 2020–2021, and to an increase of 27% in suicides in Canada (37, 38).

Besides the economic burden, many other factors contribute to increased risk of suicide in the COVID-19 world. As already mentioned, people have been highly exposed to information regarding the COVID-19 crisis through media, predominantly negative information—death statistics, cases increase, speculation about more restrictive measures, predictions and models about future unemployment and economic and sanitary crisis, among others. This unlimited exposure proves to be deleterious on many levels. People are presenting a distorted perceptions of risk, mostly of increased risk, which raises the levels of fear and apprehension (39). Furthermore, people are experiencing several non-bereavement related losses—namely loss of social connections, of their role in the professional and social milieus and of daily routines. This may trigger a grieving like reaction, particularly when these losses markedly impacts one's sense of identity (40). Additionally, social disconnection and physical distancing may induce feelings of loneliness and potentiate social isolation. All these factors lead to rumination about the current world situation and the future, placing people at risk for increased feelings of hopelessness and helplessness (4). As Beck et al. (41) reported, hopelessness is a better predictor of suicidal behavior (both fatal and non-fatal) than depression. Feelings of hopelessness, helplessness, and rumination, associated with the predisposition for negativity bias, makes individuals prone to depressive thinking (42). This may trigger the evolution from sadness to depression. While sadness is a normative emotional response to an unfavorable context, encompassing several personal losses and external stressors, depression is a pathological response. Depressed individuals perceive losses as irrevocable (e.g., there is no way the world is going to survive this pandemic, or the economy will never be restored), increasing the overwhelming feelings of lack of hope for the future and that no action and no one will be able to help them or change the current situation (4). An associated loss of purpose may ensue, since individuals find personal sources of reinforcement in activities such as work, social groups, family reunions and leading roles in daily routines (4). This feeling of meaninglessness in the context of uncontrollable circumstances, with no end at sight, further aggravates situations of depression and suicidal thoughts.

Several authors tried to identify the main groups at risk for self-harm/suicide, having highlighted five specific populations: (1) older people (43); (2) individuals with previous mental health problems (44); (3) homeless (45); (4) migrant workers (46); and (5) healthcare workers (47).

Elder people are a population vulnerable to several environmental factors—from the increased healthcare needs (with frequent medical appointments, medication and possible dependence from a third person), to the lack of social and familiar support, low income, and fragile housing conditions. The pandemic context potentiates these vulnerabilities—with suspension of home support services, scheduled medical appointments and family reunions—promoting feelings of disconnectedness from society, devaluation and burdensomeness (48, 49). This constellation of factors and feelings largely potentiates symptoms of depression and anxiety, making elder people at risk of self-harm/suicide. People in this age group (>65 years old) are a special cause of concern, due to the fragile equilibrium between protecting them from the infection and possible clinical severity of COVID-19 consequences, and assuring the social, medical and emotional support they need to go over such difficult times.

Mental healthcare has been a worldwide concern for several years. Even before the pandemic, it is estimated that about 50% of people needing mental health services in developed countries lacked access, a figure that reaches 90% of cases in low income countries (50). Research done after the 2003 SARS outbreak has shown that, 1 year after the outbreak, survivors presented concerning levels of depression, anxiety, and stress, with about 64% reporting probable psychiatric disorders (51). The context of COVID-19 pandemic poses increased obstacles to mental healthcare provision: first, services are not equipped and prepared to face the expected increase in demand; second, due to recommendations of social distancing, mandatory lockdowns, diversion of healthcare resources to COVID-19 cases and fear of being infected, scheduled appointments are either being canceled or people not attending them. Furthermore, psychiatric decompensations and suicidal thoughts may be undervalued or not recommended to present to the Emergency Departments (52). Community mental health support has also been hampered by the pandemic context: face-to-face appointments canceled or replaced by phone or video calls, doctors overwhelmed by follow up of COVID-19 affected patients and tracking of contacts, patients unable to renew prescriptions and unable to contact medical support in cases of emergency. Regarding phone and video calls, many patients felt several restrains to fully expose their current status to the clinician—lockdowns, home quarantine and lack of a personal space, frequently with other family members being able to follow the consultation, hindered the quality and assertiveness of indirect follow up. This inability to adequately follow previously diagnosed patients adds to the expected increase in mental health problems.

Evidence from countries struck earlier by the COVID-19 breakout point toward a significant increase in mental health problems and needs. A study by Wang et al. (53) described that the population of 194 provinces in China presented high levels of moderate to severe anxiety (28.8%), moderate to severe depression (16.5%) and moderate to severe stress (8.1%), with females and students being the most affected. Another research article from Iran highlighted the increased levels of stress and mental morbidity, underlining the role of unpredictability, uncertainty, seriousness of the disease, misinformation and social isolation as the factors that most contributed to it (54). Shigemura et al. (47) described the high risk of maladaptive and disruptive behaviors, such as hoarding and stockpiling of resources, in the context of high levels of fear and panic.

Several authors raised concerns about the capability of the healthcare system to answer the increased needs of mental healthcare that will come after the pandemic. Marques et al. (8) transposed the need to flatten the infection curve to the reality of mental health—there is an imperative need to flatten the mental heath demand curve, because services will not have the means to provide the care needed.

Healthcare workers are another vulnerable group for the development of mental health disorders and increased suicide risk. As previously exposed, hospitals and clinics are overloaded with work, trying to care for COVID-19 patients while simultaneously attending non-COVID-19 disorders—many of which were let without any kind of healthcare services during the peak of the first pandemic wave. Due to the increased workload and the fear of infecting their loved ones, clinicians are isolating themselves from their families, neglecting their own selfcare, personal space and individual needs (55, 56). Moreover, clinicians are under intense ethical and moral dilemmas, since in many services they are entitled to decide who will have access to intensive care therapy and who does not (55). This awareness regarding the reality of the disease course and of the fragilities of clinical practice, boosts the fear of becoming severely ill and having to arrange who to provide for their families. A cross-sectional survey including 1,257 healthcare workers from China described that 50.4% of responders were experiencing high levels of depression, 44.6% increased levels of anxiety, 34% insomnia, and 71.5% impacting distress (57). Healthcare workers are at risk of a particular type of trauma, which is secondary traumatic stress—the stress response occurring as a result of helping someone who is experiencing trauma, characterized by excessive worry and fear, high alert levels, rumination and intrusive thoughts and physical signs of stress (58). In the present context, we can add moral stress to this equation—the physical or moral distress experienced when internal or external limitations prevent someone of acting the way he believes is right (59). It is intimately associated with burnout, which may bring about feelings of exhaustion, depersonalization and possibly a dehumanization from their action (60). All these personal a professional factors make healthcare workers at high risk for psychiatric disorders and suicide risk.

As a final note, it is important to highlight the increased risk survivors of COVID-19 have to suffer from mental health disorders and suicidal thoughts/consummated suicide (61) (see for review). Patients recovering from COVID-19 usually have had physical symptoms for a long time and face psychosocial difficulties such as professional and financial problems. As already described in the literature, both physical symptoms and psychosocial factors contribute to suicidal behavior (62). In these individuals, the single most significant predictor of suicide is the presence of depression. Recovered COVID-19 patients should be followed-up and screened for both depression and suicidal ideation (61). In fact, many survivors of the new coronavirus disease will probably need long-term psychological interventions. Thus, there is an imminent need to implement specific strategies to ameliorate the psychological condition of COVID-19 survivors and to reduce the incidence of consummated suicide in this population.

## COVID-19 Suicides

Since the beginning of the COVID-19 epidemic, posteriorly classified as pandemic, several case reports of suicide have been published. In Bangladesh, there have been at least eight COVID-19 suicide cases (63). Identified triggers for these cases were COVID-19 fear, xenophobia and economical related issues. In India, two suicides were reported: one of a man convinced of being infected with Sars-CoV2, who isolated himself in order to protect his loved ones and later commited suicide; another of a man admitted to the isolation ward of the Safdarjung Hospital, from where he jumped off the window, ending his life (64). In the USA, there was the report of a murder-suicide in Chicago, where a man killed himself after killing a woman due to the conviction that both of them were infected (65). In Germany, the Hesse State Finance Minister committed suicide due to the concerns about the future, uncertainty, feelings of hopelessness and worthlessness (66). In England, a nurse working at the King's College Hospital in London ended her life while treating COVID-19 patients (67). In Italy, one of Europe's countries most hardly struck by the first pandemic wave, national media described at least five suicides during the period from February to April 2020 (68).

Another kind of suicide described in the literature is suicide pacts between couples. Griffiths el al. (69) described the self-inflicted death of 6 couples from four countries, in the context of the COVID-19 pandemic.

Thakur and Jain (70) reviewed published suicide reports, having organized suicide triggers in four categories: (1) social isolation/distancing; (2) worldwide lockdown creating economic recession; (3) stress, anxiety and pressure in medical healthcare professionals; (4) social boycott and discrimination.

## Complicated Grief in Suicide Survivors the Pandemic Context

Grief is an adaptive process that occurs after a significant loss and comprehends emotional, cognitive, physical an behavioral responses (71). Although mostly associated with the loss of a affectively close person due to death, people are subjected to multiple significant losses in their daily living, particularly in the pandemic context—loss of their health, of financial security, loss of social and affective connections, loss of freedom to move and to plan their close future (55).

Nowadays, grief is described as a two stage process, comprehending acute and integrated grief (72) (see for review). Acute grief is characterized by an intense emotional expression and predominance of negative affects, with disbelief, sadness, anxiety, disturbed sleep, excessive preoccupation and intrusive images of the deceased person, as well as an intense felling on longing and desire to be close to the lost loved one. This acute phase also encompasses a decrease in functionality in all areas of daily living—personal, social and professional. As the grieving process evolves, the individual progressively adapts to the new reality, restructuring his daily routine, social life and future projects, with negative affect and thoughts regarding the deceased diminishing in intensity and impact. This transition period usually lasts for 6–12 months. However, in the presence of internal or external disturbances, the evolution to integrated grief and the functional recovery may not occur. This persistence of acute grief manifestations has been designated complicated grief. Symptoms of complicated grief occur in up to 10% of grievers in the general population and are associated with significant psychiatric and medical morbidity (73). Complicated grief increases the risk for cancer, cardiac events, sleep disturbances, alcohol and substance abuse, major depression, anxiety disorders and self-harm behaviors, including suicide (74).

There are several identified risk factors for the development of complicated grief, including how intimate the relationship was, the degree of kinship, the type of death (for example, traumatic or sudden death), inability to say goodbye or to perform culturally approved mourning traditions, among others (75). In the world of COVID-19, many of these risk factors are affecting people worldwide. People are dying alone (either due to physical distancing or due to hospital admission) and unable to communicate with their loved ones; death and mourning rituals have been suspended and burials are being performed in mass graves and by military forces in many countries (49, 68). Dying in the pandemic context has been compared to death in the context of natural disasters, due to the elevated number of deceased people, marked unpredictability and high impact of collateral damage observed. Establishing a parallel with natural disasters, people grieving in the setting of COVID-19 pandemic, present increased baseline worry and fear due to the risk of losing their own health an, possibly, their own life (76). People grieving loved ones who lost their lives to natural disasters also present significantly higher levels of complicated grief (77).

Suicide is, by itself, a risk factor for the development of complicated grief—a sudden death, usually traumatic, many times discovered by a close contact. In fact, it has been described that more than 60% of people grieving suicide present complicated grief symptoms (74). Mitchell et al. (20) described that closer relationships to the deceased are related to higher incidence of complicated grief in suicide survivors—particularly spouses, parents and children. This should be a topic of concern to the healthcare staff, in order to provide close follow-up and adequate support this vulnerable group.

High levels of pathological grief symptoms may even persist several years after the death and be associated with comorbid post-traumatic stress disorder—an item particular to suicide related grief (78). The risk of developing PTSD symptoms increases if death occurred by violent methods or if the family member witnessed it or found the body (12). Complicated grief in the population of suicide survivors has also been associated with increasing levels of depression and hopelessness, fewer positive future perspectives, lower perceived life satisfaction and higher suicidality risk (74, 79). Shear and Prigerson concluded that suicide survivors presenting complicated grief presented twice the rate of recurrent and current depression, when compared to individuals presenting complicated grief by other types of death (80). In a study by Latham and Prigerson suicidality remained increased during a 4 month follow up period, adding to the abovementioned risk factors for the development of psychiatric disorders in suicide survivors (79). Individuals grieving suicide are, in fact, at increased risk to attempt and complete suicide (18).

Regarding the cognitive and emotional reactions of people grieving suicide, they usually present intense shock or disbelief, greater levels of guilt, shame, anger, as well as feelings of rejection and abandonment (81). Additionally, most of them persistently experience the “agonizing questioning” about why the death occurred, why the person did not found other solution, and why they were not able to predict or help (82). One of the major obstacles for people bereaving suicide to overcome it, and if necessary ask for help, is the objective and perceived stigma. Stigma against suicide survivors comprehends the inclusion into stereotypes, the prejudice and discrimination (83), being considered pervasive and persistent (84). The internalized dimension of stigma poses a major hurdle to accede specialized help: it triggers feelings of devaluation, shame, need of secrecy, and tendency to apply negative stereotypes to oneself (85, 86). Perceived stigma is particularly harmful to people with high levels of interpersonal sensitivity, hostility and paranoid ideation, since it potentiates increased levels of psychological distress that intensifies along time (87).

Suicide amidst a pandemic poses as a double traumatic event for suicide survivors. People are trying to adjust to the contingencies of this new reality, already prone to psychological challenges, managing to maintain the healthiest routine possible and support their friends and family. Being struck by the suicide of a loved one may raise the levels of confusion, doubt, non-acceptance, hopelessness and helplessness to unbearable levels, making people mentally ill. At the present time, stigma is an important factor to be addressed. People are yet trying to handle the stigma of Sars-CoV2 infection—due to the tight sanitary measures and focus on individual responsibility, infected people are seen as uneducated and careless. Besides putting a hard toll on those diagnosed with the infection, this increases the feelings of guilt and shame, that may potentiate psychological distress. In this context, the death of a loved one to suicide may be handled as a double loss and a double picture of stigma—first, the loss of security and health, then a loss by death. This potentiates the appearance of negative outcomes and intensifies the urge to educate society about suicide and promote the adequate follow up of suicide survivors.

## Interventions

Regarding the theme of preventing complications and intervening in the grieving process of suicide survivors, first it is important to approach some strategies to prevent suicide. In terms of direct suicide prevention interventions, there is increasing evidence for multi-level systemic approaches—using components ranging from individual post-traumatic care (for example, assertive post-traumatic care, psychosocial interventions) to public health interventions (e.g., targeting general risk factors, population education on suicide, general practitioners training) (88). While the increase in telehealth services is critical, it is unlikely that the health professionals available to support them will increase to meet the needs, and services that include automated digital components may be a more efficient solution (89). As described above, people at increased risk for suicide present a number of cognitive and perceptual distortions, that can be addressed through psychotherapy. In the presence of an increased risk perception, the therapeutic objective is to help an individual to develop an accurate risk perception so that their fear is proportional to the threat. The primary strategy to be used is cognitive restructuring (39)—to identify, examine and, where appropriate, reassess the situation. Controlling stimuli to combat feelings of despair is also recommended. Although not helpful, paying attention to negative news in the media is in our nature and checking the news often can add to despair, even in the hope of more positive adds (4). Regarding non-grief losses, expressive writing in the sense of identifying and naming the loss experienced can help individuals to increase awareness of associated emotions and recognize previous strategies that have been effective in dealing with that emotion in the past. After naming losses, outlining ways to advance or change these areas of loss can help individuals to accept their loss and work to reproduce what has been lost. Useful strategies that can be used to think about current regulations in a more balanced way include: re-marking the current guidelines from “social distance” to “physical distance,” remembering that individuals are now separated so that they can be together later; and reshape the situation as a time to focus, build and / or create meaningful relationships (4).

Although there is a dearth of treatment studies in suicide survivors, experts agree that: (1) initial attention should be focused on traumatic distress; (2) self-help support groups can be beneficial; and (3) there is a role for both pharmacotherapy and psychotherapy in those who already show adverse effects on mental health or at high risk for serious and persistent difficulties (90). With regard to the symptoms present in suicide survivors, some longitudinal studies have shown that women are at greater risk of depression, but not of complicated grief, after a loss by suicide than men (91). Prigerson et al. (73) showed that the symptoms of complicated grief can also be debilitating in the absence of a depressive component, although the two commonly coexist.

Pharmacotherapy has played an important role in dealing with the presence of different symptomatic expressions and different gender variations. However, it still lacks a consistent amount of empirical studies, thus leaving doubt about its effectiveness. Contrary to the symptoms of reactive depression in grief, the symptoms of complicated grief may persist after treatment with tricyclic antidepressants (74). Pharmacotherapy can reduce depressive or anxious symptoms during bereavement, but psychotropic drugs cannot help a bereaved person in difficulty as to the personalized meaning of their loss (92–95). Focusing on individual antidepressant drugs, the literature suggests a preliminary promise for the use of bupropion (93) and escitalopram (96). In conclusion, given that there is some evidence that the use of psychiatric drugs can be beneficial in complicated grief in situations not related to suicide, pharmacotherapy can also be useful for survivors of suicide with complicated grief.

More than four decades ago, Shneidman (97) identified the provision of adequate support for suicide as a major public and mental health challenge. Postvention has become available in an increasing number of countries, and has been recognized as an important early intervention strategy in the mourners for suicide, designed to promote active follow-up and to avoid the development of psychopathological complications (98). As for the role of psychotherapy in addressing this issue, psychotherapeutic interventions to date have been designed to reduce the symptoms of complicated bereavement disorder since its inception. The treatment modalities that have proven effective include structured writing tasks (99) interpersonal psychotherapy (100), therapy for complicated grief (101, 102), and cognitive behavioral approaches transmitted face to face or via the Internet (103, 104), all of which rely on professional intervention.

Although self-help groups or counseling services for suicide survivors in the voluntary sector are steadily increasing, only a few psychotherapeutic interventions for survivors after suicide have been developed and scientifically validated. A recent systematic review included a total of 11 studies on treatment of suicide grief; however, only few of the included studies have shown evidence of efficacy in helping people suffering from uncomplicated bereavement, and empirical evidence is still lacking for interventions aimed at complicated grief disorder after suicide [for review see (105)]. Despite not being specific to grief suicide, studies support the use of cognitive behavioral therapy (CBT) (106), limited interpretive group therapy in time (107), and complicated grief therapy. Complicated grief therapy (CGT) is a modification of interpersonal psychotherapy, adding elements of cognitive behavioral therapy, exposure, gestalt and motivational interviewing. The basic principle underlying CGT is that acute grief will instinctively transition to integrated grief if the complications of pain are addressed and the natural grieving process is supported. Each session includes grief work focused on losses, as well as attention focused on restoration. Studies support the robust efficacy of CGT for the treatment of complicated grief, even in severe and chronic situations, as well as in the presence of comorbidity (108). When complicated grief occurs in the context of grief suicide, the psychiatric and psychological literature provides few, if any, empirical guidelines based (109). It is not unlikely that the abovementioned CGT may be beneficial for many CG suicide survivors, but therapy may need to be modified to give more emphasis to the recurrent themes of suicide grief: the quest to understand why, the feelings of guilt, rejection, shame, anger and the stigma.

It has also been explored whether a telephone intervention providing education and support from trained volunteers could be a cost-effective intervention to prevent complications—such as major depressive disorder or complicated grief—in the first year post suicide. During the first 6 months, the grief-related investigation did not value the scores on the grief severity scale, since they fluctuate as the acute process evolves. Authors speculate that it is precisely during this period that the complications of grief can be more effectively prevented with a supportive intervention. The primary result for this pilot proof-of-concept study shows that recently suicide grieving subjects could be recruited and retained for up to 13 months after the loss using this approach (110). These results point toward a possible alternative for an initial contact approach, given the restrictions imposed by the pandemic context. A committed, humanistic and compassionate approach would be preferable to overly directive or passive approaches. Other studies showed that an assessment addressing grief reactions and the creation of suicide narratives were crucial therapeutic aspects (111). For most suicide survivors, participation in support groups is a way of feeling understood, a place where feelings are accepted—providing a mean for catharsis. Successful support groups have common components, which include accurate information, permission for suffering, normalization of effects and behaviors that are not in line with the individual's normal state and above all, support in the process. Observing the suffering of other people who go through the same process, can be useful in helping others.

Schleider et al. (112, 113) developed and tested brief and accessible interventions, provided in non-traditional environments. They found that a single session of a solution-centered consultation was associated with a reduction in psychological distress in adults seeking psychotherapy.

Considering the growing need to prevent suicide in the context of a pandemic, on the one hand, as well as to act on suicide survivors, on the other, it is necessary to improve the access to mental healthcare, given the expected shortage of providers care (8). When organizing psychological assistance within and at various stages of the pandemic, Inchausti et al. highlight four main challenges (114): (1) deficiencies in healthcare systems—health structures are lacking both technicians and material, including mental health professionals specialized in the psychological approach to crises and emergencies (most health professionals are not trained neither in crisis and catastrophe intervention, nor in grief support for bereaved individuals); (2) the lack of knowledge of the general population, and even of health care technicians, regarding both short and long term psychological consequences of pandemics and, consequently, limited effort to provide resources to deal with them. (3) lack of structure, team hierarchy and planning of psychological interventions, particularly if applied at different levels and by professionals from different classes (from primary care services to specialized mental health professionals)—a correct articulation is essential for an assertive treatment and adequate continuity of care, even after the acute phase of the pandemic; (4) in emergency contexts, there is always a risk associated with initial unstructured responses to the crisis, related to rapidly implemented interventions and structures associated with an excessive supply of well-intentioned but potentially not evidence-based psychological assistance (114).

In a world full of restrains, support lines and teleconsultations are an effective and necessary alternative, especially in times of crisis. Helplines were created to resolve, or minimize a crisis situation, in a time of crisis, and not to resolve a long-term situation, providing advice, referral and follow-up. In a study published by Oliveira et al. (115) it is said that assistance in the emergency line is an asset in several ways, in that it helps to save lives in the negotiation of suicide and acceptance of help, and provides emotional stabilization and counseling to many requests for psychiatric help (116).

A review of recent studies focused on the effectiveness of “telepsychological” interventions in clinical populations of adults with emotional disorders has shown that these interventions are promising. Different types of interventions using communication technologies have been reported in the literature, evidencing their effectiveness in providing mental health services in general and in epidemic situations in particular (117).

Internet-based interventions for symptoms of grief proved to be effective, with moderate to high impact that could be maintained over more than 1.5 year follow-up, with the largest effect being noticed in symptoms of PTSD (118). Despite being a population with high risk for psychiatric complications, as already mentioned, suicide survivors do not often look for professional help (119). Thus, the anonymity provided by the online environment may facilitate their access to treatment, decreasing aspects such as negative psychosocial attention or fear of stigmatization, and promoting an earlier intervention. In the context of home lockdown, as well as in countries with scarce access to specialized health care, Internet-based interventions also allow for a wider access to treatment, regardless of geographic location or transportation means. In a progressively more online society, internet-based psychotherapeutic interventions present as a captivating and flexible approach.

Most internet-based grief interventions were based on an individual setting and usually include several types of writing tasks—from more extensive and structured writing tasks. Classical grief group interventions provide many advantages to the participants, bringing to the setting important therapeutic elements—e.g., universality of suffering, group cohesion, behavioral model learning and interpersonal learning (120, 121). Regarding the online setting, studies of group interventions have been performed mainly through videoconferencing. Wagner et al. (105) studied the applicability and efficacy of an online group intervention based on CBT in individuals grieving by suicide, using webinars. Webinars and videoconferencing are the online interventions that show results more similar to traditional group interventions for people bereaved by suicide (122). The webinar intervention format was chosen to allow for simultaneous real-time interaction between the therapists and participants groups. Furthermore, webinars have the added advantages of allowing the display of videos and psychoeducational presentations in PowerPoint, as is usual in e-learning environments, and participants can remain largely anonymous, which can diminish the restrains to seek professional help. This intervention strategy led to clinical results similar to the ones of face-to-face interventions, posing as a psychotherapeutic alternative in the COVID-19 pandemic context (122).

## Conclusion

Our world has been overwhelmed by the COVID-19 pandemic. Most of the information regarding the possible impact on populations of the pandemic on populations were discussed and assemble at the peak or toward the end of the first wave of the COVID-19 pandemic. At the time of the writing of this article, Europe and the United States of America are already under the second wave. High organizations estimate that restrictive measures might be necessary for at least 6 months to 1 year more. The economy is on decline and not having the time or opportunity to recover. The health care system is still rebuilding itself from the first wave, while trying to have better conditions to face the new surge of COVID-19 cases, as well as to answer people with other diseases, namely mental health disorders. The high risk groups mentioned above are progressively more vulnerable to the social, economic and personal constrains of the current times. Light should be shed over these populations in order to prevent further development of mental disorders and the loss of more lives. It is mandatory that mental healthcare structures are reinforced, psychological helplines restructured and multidisciplinary teams assembled and hierarchically organized. Online support and psychotherapeutic intervention is an available and effective approach. Thus, health technicians have to adapt to the reality of distant intervention and application of new technologies, in order to reach those in need in the COVID-19 world. Suicide survivors have to be actively reached for and it starts with healthcare personnel—those who contact with the suicide victim should actively signalize close contacts to psychological support teams, in order for them to feel adequately supported and to enable early intervention whenever needed.

## Author Contributions

All authors contributed to the bibliographic review, structuring, writing, and revision of the manuscript.

## Conflict of Interest

The authors declare that the research was conducted in the absence of any commercial or financial relationships that could be construed as a potential conflict of interest.
