# Erratum: COVID-19 Suicide Survivors—A Hidden Grieving Population

**DOI:** 10.3389/fpsyt.2021.653544

**Published:** 2021-02-08

**Authors:** Frontiers Production Office

**Affiliations:** Frontiers Media SA, Lausanne, Switzerland

**Keywords:** complicated grief, suicide, suicide survivors, COVID-19, intervention, prevention

Due to a production error, there was an error in affiliation for authors Sara Pinto, Alzira Silva, Rosário Curral, and Rui Coelho. Instead of “Psychiatry Service, Centro Hospitalar Universitário São João, Oporto, Portugal,” it should be “Psychology Service, Centro Hospitalar Universitário São João, Oporto, Portugal.” Only author Joana Soares is affiliated to “Psychiatry Service, Centro Hospitalar Universitário São João, Oporto, Portugal.” The publisher apologizes for this mistake.

The original article has been updated.

