# Erratum: COVID-19 Suicide Survivors—A Hidden Grieving Population

**DOI:** 10.3389/fpsyt.2021.793655

**Published:** 2021-11-02

**Authors:** Frontiers Production Office

**Affiliations:** Frontiers Media SA, Lausanne, Switzerland

**Keywords:** complicated grief, suicide, suicide survivors, COVID-19, intervention, prevention

Due to a production error, there was an error in the numbering of the affiliations. The corrected affiliation list appears below.

Sara Pinto^1,2*^, Joana Soares^3^, Alzira Silva^1,2^, Rosário Curral^1,2^ and Rui Coelho^1,2^

^1^ Psychiatry Service, Centro Hospitalar Universitário São João, Oporto, Portugal

^2^ Department of Clinical Neurosciences and Mental Health, Faculty of Medicine of the University of Oporto, Oporto, Portugal

^3^ Psychology Service, Centro Hospitalar Universitário São João, Oporto, Portugal

The publisher apologizes for this mistake. The original version of this article has been updated.

